# 0D Dynamic Modeling and Experimental Characterization of a Biomass Boiler with Mass and Energy Balance

**DOI:** 10.3390/e24020202

**Published:** 2022-01-28

**Authors:** Fateh Mameri, Eric Delacourt, Céline Morin, Jesse Schiffler

**Affiliations:** 1 CNRS, UMR 8201–LAMIH, University Polytechnique Hauts-de-France, 59313 Valenciennes, France; fatehmameri@hotmail.fr (F.M.); Eric.Delacourt@uphf.fr (E.D.); 2 INSA Hauts-de-France, 59313 Valenciennes, France; 3 CNRS, UMR 7357–ICube, University Strasbourg, 67412 Illkirch, France; schiffler@unistra.fr

**Keywords:** energy balance, biomass boiler, heat exchanger, 0D modeling, Bond Graph, global thermal transfers, inverse method

## Abstract

The paper presents an experimental study and a 0D dynamic modeling of a biomass boiler based on the Bond Graph formalism from mass and energy balance. The biomass boiler investigated in this study is an automatic pellet boiler with a nominal power of 30 kW with a fixed bed. The balances allow to model as time function the flue gas enthalpy flux variation and the thermal transfers between the flue gas and the walls of the boiler subsystems. The main objective is to build a model to represent the dynamic thermal behavior of the boiler. Indeed, small domestic boilers have discontinuous operating phases when the set temperature is reached. The global thermal transfer coefficients for the boiler subsystems are obtained according to an iterative calculation by inverse method. The boiler has an average efficiency of 67.5% under our operating conditions and the radiation is the dominant thermal transfer by reaching 97.6% of the total thermal transfers inside the combustion chamber. The understanding of the dynamic behavior of the boiler during the operating phases allows to evaluate its energy performances. The proposed model is both stimulated and validated using experimental results carried out on the boiler.

## 1. Introduction

Biomass plays a significant role in the development of clean and sustainable heat production processes with a large reduction of CO_2_ emissions [1]. There are multiple ways to exploit energy potential of biomass, e.g., by pyrolysis [2], gasification or other bio-chemical processes using bacteria to generate gaseous and liquid biofuels or by direct combustion to generate heat and electricity [3,4,5]. Even if biomass has a lower calorific value than other fuels, such as fossil fuels, this source of energy remains cleaner with some reserves [6]. The biomass can be valued for the simultaneous production of heat and electricity from CHP (Combined Heat and Power) plants [7,8].

In the thermal conversion of biomass, there are multiple physical and chemical processes that have an influence on the performances of industrial and domestic applications, such as furnaces, industrial burners and biomass boilers [9], the exergy analysis must be used in order to find the best way to recover the maximum of mechanical work in a CHP (combined heat and power) unit. Biomass boilers provide a direct conversion of biomass into energy by combustion. They are widely investigated in several configurations according to delivered power: biomass domestic boiler of 24 kW, 27 kW and 32 kW [10,11,12], industrial biomass boiler of 4 MW [13].

Dynamic modeling of energy systems can be used for the design, the optimization or the control of the studied process. Tognoli and Najafi [14] provided a detailed dynamic model of an industrial fire-tube boiler with five different geometrical configurations. The dynamic model developed consists of two main sections separated on the flue gas side and the evaporating shell. Both sides are integrated employing an energy balance. Then, a PID tuning was implemented for each boiler to control the vapor pressure, while responding to a demand with variable mass flow rate. The operation of the boilers was simulated to meet four different steam demand profiles. A wood pellet micro-cogeneration system with steam engine was modeled by Bouvenot et al. [15] and implemented in the TRNSYS code. Both theoretical and experimental approaches have been adopted to develop the model. The authors presented the dynamic response of the installation and took into account the steady and transient states. A dynamic model applied to two biomass boilers with nominal power of 6 and 12 kW was presented by Carlon et al. [16]. The model developed with TRNSYS calculates the mass and energy balances of the boilers under time variable inputs. It describes the operation of the boiler under dynamic conditions and provides the chemical composition of the flue gases from the chemical composition of the wood pellets and the value of the excess air and by adopting the assumption of a complete conversion of the mass of fuel. The model has been tested for two modes of boiler operating conditions: full and variable load and steady and transient states. The results of the modeling showed a better agreement with the experimental data during steady operation as well as in dynamic mode.

The modeling of thermofluidic systems related to heat and power generation are also described in terms of mechanical work generation processes. We can note for example, a study on the modeling of ORC (Organic Rankine Cycle) systems investigated by Ziviani et al. [17]. The authors presented an overview of the problems related to ORC modeling and developed an efficient and powerful simulation for an ORC system adapted to the exploitation of low-grade thermal energy. Other physical systems are modeled like ECE (External Combustion Engine), for example Stirling or Ericsson engines [18,19,20]. Due to their promising future paths for energy cogeneration by coupling them with thermodynamic systems, small biomass boilers were also studied from this type of approach. However, they raised several concerns, ranging from design to dynamic control [21]. Inappropriate power requirement definition and inadequate control can affect the boiler performances and reduce its efficiency. To facilitate the design process and overcome upstream design failing, the modeling represents a very interesting approach.

The dynamic behavior of this kind of system is generally described by non-linear differential equations. A suitable method, as the Bond Graph formalism, is necessary to well understand physical interactions in a such thermofluidic system. Therefore, an appropriate model that represents a system involving energy transfers can be extracted in a structured way [22]. The Bond Graph method is based on a graphic structure representing the power exchanges between different physical entities considered in multidisciplinary dynamic systems. It was initiated by Paynter in 1961 [23] and then developed by Karnopp and Rosenberg [24]. This tool is adapted to the modeling of the physical processes involved in different energy fields (hydraulic, mechanical, electrical, chemical and thermal). Bond Graph formalism allows to develop a parametrized model with an unified language that interprets the power transfers within the system considered explicitly through its graphic structure. Bond Graph investigations have been carried out on energy systems such as hot air engines (Ericsson engine [19]), Heating, Ventilation and Air-Conditioning (HVAC) systems [25], industrial biomass boiler [26], endoreversible heat engine [27], thermo-hydraulic system [28] and in chemical engineering [29]. Ould-Bouamama et al. [30] have developed a dynamic model using Bond Graph methodology for an industrial chemical reactor. The purpose of this application is to design a monitoring and survival platform in case of failure.

The modeling of biomass boilers operation has been the subject of several studies. Mathematical models based on thermodynamic laws have been developed to represent the dynamic behavior of the boilers during operating phases such as start-ups and load changes [31]. Åström and Bell [32] developed a simple non-linear model based on the first law of thermodynamics and configured with the basic design data of the boiler. Sandberg et al. [33] presented a dynamic model based on the mass and energy balances of a biomass boiler to study the effect of fouling on boiler performances. Table 1 summarizes the experimental and numerical studies of the literature about different systems (boiler, furnace, reactor and engine).

Published studies on the dynamic modeling of boilers often refer to black box or grey box models. There are some studies describing white box models but the detail of the modeling is often incomplete (use of components of commercial tool libraries rather opaque or description of physical phenomena modeled without specifying the interactions between them).

In this work, a 0D dynamic model of a domestic biomass boiler is provided using the Bond Graph formalism to simulate its dynamic behavior and to understand all the heat transfers involved in the boiler. The 0D model is based on mass and energy balances. It characterizes all the heat exchanges between the flue gas and the walls of the subsystems constituting the boiler. This dynamic modeling makes sense with domestic boilers whose operation is typically discontinuous unlike larger industrial boilers. The thermal needs of the house are variable which results in intermittent operation of the boiler. The strength of dynamic zonal modeling is to be able to predict the time evolution of different state variables of a complex system by coupling some fields of physics (mechanics, thermodynamics, …). Moreover, it is possible, during the simulation, to insert time boundary conditions from in-situ measurements. The local evolution of the state variables is much less detailed than with CFD modeling but the dependencies of one zone with another are better taken into account with a 0D dynamic modeling. Moreover, CFD simulations are generally performed in steady state (averaged) because of the high computational cost in unsteady state, contrary to the dynamic 0D model which is able to predict the impacts on coupled systems. CFD and dynamic 0D modeling are therefore to be implemented according to the targeted objectives and the simulations results can hardly be compared. However, they can be efficiently coupled in multi-scale approaches. The objective of this study is to model the dynamic behavior of the boiler during the operating phases in order to take into account the variability of the heat production with regard to the thermal load of the heating network or of any system which could be connected to it (hot air machine for example in the case of a CHP plant).

Compared to other dynamic modeling, the interest of Bond Graph methodology by its explicit graphic structure is to make clearer the modeling process of coupled multi-physical phenomena with blocks linked together by power links where effort and flow variables as well as causality are explicit. This methodology is very well adapted to model a system with thermal and mass transfers described with linear or non-linear differential equations.

The Bond Graph formalism was not developed in the literature to study the thermal transfers between the different fluids in a low power biomass boiler during the transient operating phases but it is increasingly used for the modeling of thermofluidic systems in general. Thanks to this formalism and its clarity, it is then possible to highlight the boiler components where the thermal transfers must be optimized and to understand the physical interactions. Moreover, there is a lack of experimental data for low power biomass boiler in the literature, these data are essential to develop a dynamic model by considering the real operating cycle of the boiler. An innovative way is used in this study by coupling experimental values and 0D modeling at each time step of the calculation with an analysis of energy performances for a domestic biomass boiler.

In the paper, the biomass boiler is described with all sensors used for the measurement of temperatures and mass flow rates. Then, the methodology is explained and the dynamic model 0D of the boiler is presented. Experimental and numerical results are discussed.

## 2. Description of the Biomass Boiler

### 2.1. Experimental Setup

**Figure 1:**
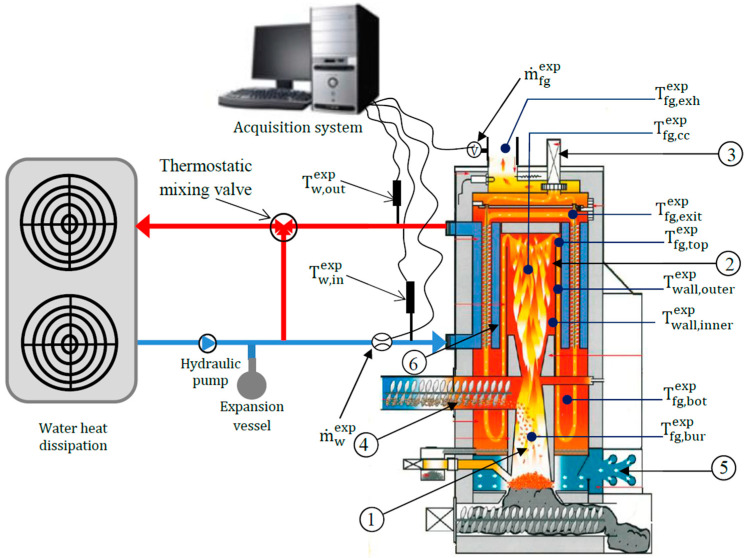
Thermocouples positions and hydraulic circuit. (1) Burner. (2) Combustion chamber. (3) Flue gas extraction. (4) Screw feeder. (5) Air inlet. (6) Water-flue gas heat exchanger.

The study is focused on an automatic domestic wood pellet boiler with a power of 30 kW Figure 1), equipped with a water-flue gas heat exchanger whose main role is to recover a part of the heat energy in the flue gas and transfer it in the water. The water circulation in the hydraulic circuit is ensured by a pump. The introduction of the pellets into the burner of the boiler is done by a screw that operates cyclically as long as the temperature of the outlet water is lower than the setpoint temperature. When the setpoint temperature is reached, the pellet supply stops. The primary air arrives through trapdoors located in the lower part of the boiler and its circulation is ensured by an exhaust fan mounted on the top cover of the boiler which is controlled by a lambda probe located in the chimney of the boiler. To dissipate the heat of the working fluid, the hydraulic circuit is connected to two air heaters located to outside of the test cell. In order to carry out an experimental characterization of the boiler, several sensors are installed at different locations in the boiler (Figure 1). An electromagnetic flowmeter with an operating range of 20 to 500 dm^3^/h with an uncertainty of 0.5% measures the water mass flow rate (${\dot{m}}_{w}^{\exp})\;$ circulating in the boiler heat exchanger. The flue gas mass flow rate $\left({\dot{m}}_{\mathrm{fg}}^{\exp}\right)$ is calculated from pressure and temperature measurement in the chimney (Pitot wing system connected to a micromanometer (uncertainty 5% and K-type thermocouple (uncertainty 0.75%) on their measurement ranges respectively). The water temperature at the inlet ($T_{w,\mathrm{in}}^{\exp}$) and outlet ($T_{w,\mathrm{out}}^{\exp}$) of the water-flue gas heat exchanger are recorded by two platinum Pt100 probes (uncertainty 0.8%). A K-type thermocouple is inserted at the chimney (uncertainty 0.75%) for the measurement of the flue gas temperature ($T_{\mathrm{fg},\mathrm{exh}}^{\exp}$). Another type S thermocouple (uncertainty 0.25%) is placed in the central axis of the combustion chamber to measure the instantaneous evolution of the flue gas temperature ($T_{\mathrm{fg},\mathrm{cc}}^{\exp}$).

K-type thermocouples (uncertainty 0.75%) are placed in the burner ($T_{\mathrm{fg},\mathrm{bur}}^{\exp}$), on the top and bottom sides of the combustion chamber ($T_{\mathrm{fg},\mathrm{top}}^{\exp}$ and ${\;T}_{\mathrm{fg},\mathrm{bot}}^{\exp}$). Two other K-type thermocouples (uncertainty 0.75%) are also welded to each side of the combustion chamber wall ($T_{\mathrm{wall},\mathrm{outer}}^{\exp})$ and ($T_{\mathrm{wall},\mathrm{inner}}^{\exp}$). A K-type thermocouple (uncertainty 0.75%) is placed at the outlet of the heat exchanger tubes ($T_{\mathrm{fg},\mathrm{exit}}^{\exp}$) (Figure 1). The flue gas temperature measurements in the burner and the combustion chamber have been corrected from radiative effects. Indeed, with such temperature levels, the radiative dissipation of the thermocouples is significant. Several methods exist to take into account this phenomenon which underestimate the true value of the temperature. The method used is the extrapolation method [35,36] which consists in using two thermocouples with wires of different diameters and therefore with different hot welds diameter (here 0.95 mm and 0.64 mm) placed at the same position. The radiative flux exchanged is assumed to be proportional to the surface of the hot weld, resulting in a zero radiative flux when the surface of the weld is infinitely small. From the two measured temperatures, an extrapolation allows to obtain the temperature value for a zero-weld surface corresponding to an absence of radiation. In our case study (in the flame and its vicinity), as an example, for a 1000 °C temperature measurement, the corrective value to be applied reach 170 °C. In this paper, the superscript “exp” corresponds to experimental measurements. The quantities calculated by the model have no superscript.

**Figure 2:**
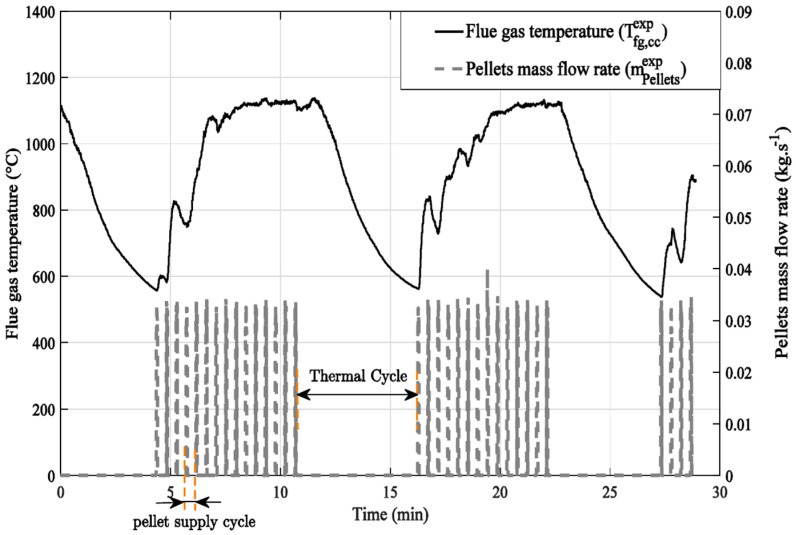
Boiler operating cycle.

The flue gas temperature evolution in the combustion chamber measured at radius of 90 mm and height of 330 mm obtained during the boiler operating cycle is plotted versus time and correlated with the pellets mass flow rate (Figure 2). It shows a strong dependence between the quantities of pellet supplied by a feed screw and the temperature increase of the flue gas in the boiler combustion chamber. The burnt gas temperature varies between 600 and 1100 °C. It increases with the arrival of pellets and decreases with their complete consumption. As mentioned above, the pellets are introduced into the boiler burner by a feed screw that rotates with a PWM duty cycle as long as the water temperature at the outlet of the heat exchanger is lower than the set temperature. This operating mode is controlled by a pulse-width modulation control. When the set temperature is reached, the pellet supply stops. Thus, a long stop of the pellet supply (12 min) can be observed in Figure 2. The pellet supply disruptions induce a drop in the burnt gas temperature in the combustion chamber with a temporary delay. The time lapse between the increase and drop of flue gas temperature defines a thermal cycle. The pellets mass flow rate is deduced from the calibration of the feed screw, according to the angular position of the screw. All sensors are connected to a data acquisition system with a dedicated code developed under Labview software.

### 2.2. Energy Balance of the Boiler

Although estimated from the rotation speed of the screw during a calibration phase without combustion, the mass flow of pellets is difficult to obtain accurately over short operating times of the screw. In fact, the quantity of pellets introduced by the screw by PWM method is not constant between each cycle because more or less large pellet clusters are detached from the screw. Moreover, the combustion is not instantaneous, it would be necessary to introduce a dynamic combustion model of solid biomass to calculate the heat release as a function of time. The boiler model presented here can be modified in the future by integrating this combustion dynamics. The pellet combustion is therefore not modelled, so the heat generated during the combustion of the pellets has been calculated using the experimental mass flow rate and the experimental temperature of the flue gases in the burner. Then, the heat flux provided by the combustion of pellets is estimated from the following equation:

(1) $${\overset{•}{H}}_{\mathrm{fg},\mathrm{bur}}\left(t\right)={\overset{•}{m}}_{\mathrm{fg}}^{\exp}\left(t\right).\left[c_{p}\left(T_{\mathrm{fg},\mathrm{bur}}^{\exp}\right).\left(T_{\mathrm{fg},\mathrm{bur}}^{\exp}\left(t\right)-T_{\mathrm{fg},\mathrm{bur}}^{\mathrm{ref}}\right)+\Delta H_{\mathrm{ref}}^{0}\right]$$

With:

$\dot{H}$_fg,bur_: heat flux released from pellet combustion (W)

${\dot{m}}_{\mathrm{fg}}^{\exp}$: experimental flue gas mass flow rate (kg.s^−1^)

$T_{\mathrm{fg},\mathrm{bur}}^{\exp}$: experimental flue gas temperature in the burner (K)

$T_{\mathrm{fg},\mathrm{bur}}^{\mathrm{ref}}$: reference temperature for flue gas in the burner (298 K)

$\Delta H_{\mathrm{ref}}^{0}$: standard formation enthalpy of gas in the burner (J.kg^−^^1^).

Considering the majority presence of N_2_ and O_2_ (air excess close to 1) in the mixture and for a first approximation, we assume that the mixture is composed as a gas including only pure species. We can therefore assume that $\;\Delta H_{\mathrm{ref}}^{0}=0$.

The energy balance of the boiler is established at each time step. It represents the heat exchanges between the flue gas and the boiler structure, the heat flux recovered by the water in the water-flue gas heat exchanger and the losses at the boiler exhaust. The outer wall of the boiler is assumed to be adiabatic because the boiler is very well insulated and the losses with the environment are negligible compared to the other heat flux. The losses are more significant from the boiler outlet through the exhaust pipe but this part is not modeled here. The heat flux released from the pellets combustion is then given by:

(2) $${\overset{•}{H}}_{\mathrm{fg},\mathrm{bur}}(t)={\overset{•}{H}}_{\mathrm{fg},\mathrm{exh}}(t)+\Delta {\overset{•}{H}}_{w}(t)+{\overset{•}{Q}}_{\mathrm{wall}}\left(t\right)$$

With:

$\dot{H}$_fg,exh_: exhaust heat flux (W).

Δ$\dot{H}$_w_: heat flux transferred to the water (W).

$\dot{Q}$_wall_: heat flux stored in the boiler structure (W).

**Figure 3:**
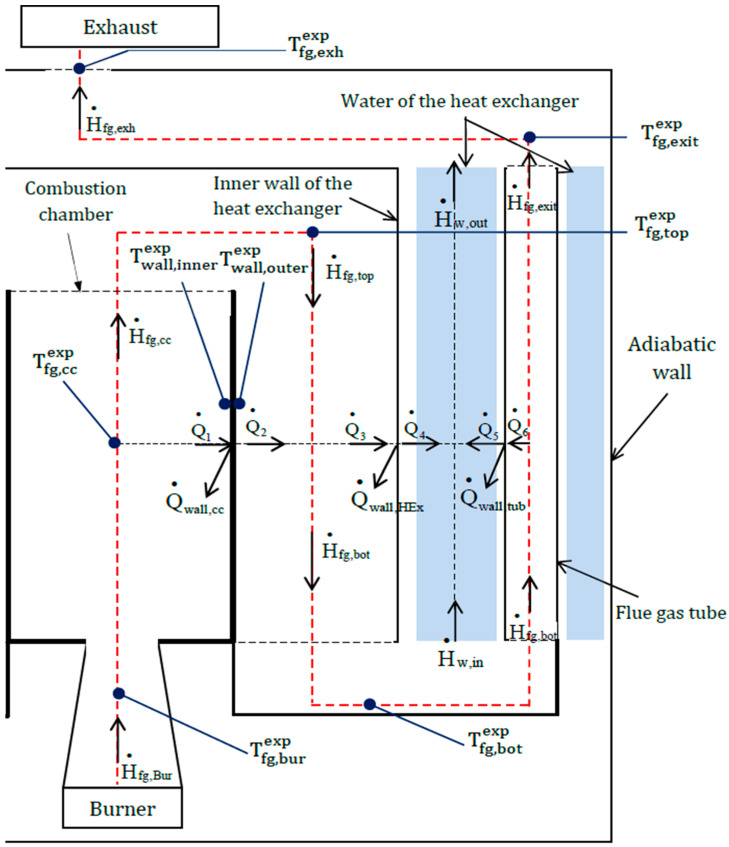
Energy balance of the boiler.

The flue gases resulting from the combustion of the pellets go through the boiler subsystems (Figure 3 dashed red line) and exchange heat with their walls. Due to the transient phases, the walls store or yield a quantity of heat flux from or to the flue gases: the heat flux stored in the combustion chamber walls ${\dot{\;Q}}_{\mathrm{wall},\mathrm{cc}}$, in the inner wall of the heat exchanger$\;{\dot{\;Q}}_{\mathrm{wall},\mathrm{HEx}}$ and in the walls of the flue gas tubes ${\dot{\;Q}}_{\mathrm{wall},\mathrm{tub}}$. The walls of the subsystems store some heat flux, consisting of three parts:

(3) $${\overset{•}{Q}}_{\mathrm{wall}}={\overset{•}{Q}}_{\mathrm{wall},\mathrm{cc}}+{\overset{•}{Q}}_{\mathrm{wall},\mathrm{HEx}}+{\overset{•}{Q}}_{\mathrm{wall},\mathrm{tub}}$$

Each of these heat fluxes is calculated in the subsystems from inlet and outlet flux (Figure 3):

(4) $${\overset{•}{Q}}_{\mathrm{wall},\mathrm{cc}}={\overset{•}{Q}}_{1}-{\overset{•}{Q}}_{2}$$

(5) $${\overset{•}{Q}}_{\mathrm{wall},\mathrm{HEx}}={\overset{•}{Q}}_{3}-{\overset{•}{Q}}_{4}$$

(6) $${\overset{•}{Q}}_{\mathrm{wall},\mathrm{tub}}={\overset{•}{Q}}_{6}-{\overset{•}{Q}}_{5}$$

The heat flux transferred to the inner walls of the heat exchanger and the flue gas tubes is partially transferred to the water.

(7) $$\begin{array}{c} \Delta {\overset{•}{H}}_{w}={\overset{•}{Q}}_{4}+{\overset{•}{Q}}_{5}-{\overset{•}{Q}}_{w,\mathrm{st}}\\ \Delta {\overset{•}{H}}_{w}={\overset{•}{m}}_{w}^{\exp}\cdot (c_{w}(T_{w,\mathrm{out}}^{\exp})\cdot T_{w,\mathrm{out}}^{\exp}-c_{w}(T_{w,\mathrm{in}}^{\exp})\cdot T_{w,\mathrm{in}}^{\exp})\\ \Delta {\overset{•}{H}}_{w}={\overset{•}{H}}_{w,\mathrm{out}}-{\overset{•}{H}}_{w,\mathrm{in}} \end{array}$$

With $\dot{Q}$_w,st_: heat flux stored by the water in the heat exchanger (W).

## 3. 0D Bond Graph Modeling

**Figure 4:**
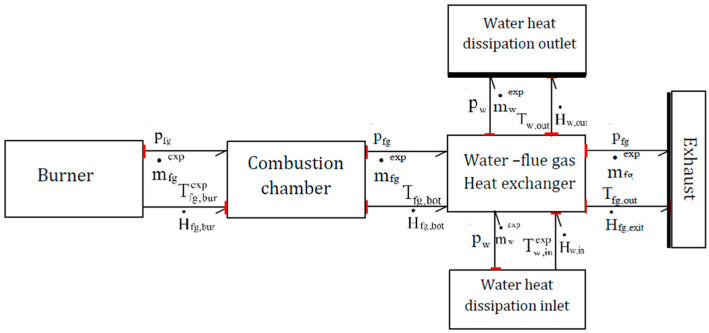
Word Bond Graph model.

The modeling of the main components of the boiler system, such as the combustion chamber, the flue gas tubes and the heat exchanger is performed using Bond Graph formalism. The boxes in Figure 4 represent the subsystems of the studied boiler, where the half-arrows characterize the thermal and hydraulic Bond Graph links between the subsystems. The word Bond Graph model describes here the thermal and mass transfers between subsystems. Causalities (**I**) are also present in order to indicate the variables at the origin of the system dynamics. In Figure 4, the combustion chamber box is not detailed, it includes the flue gas path from burner to the bottom of the heat exchanger. The combustion chamber temperature noted T_fg,cc_ is located inside this box but not appears in the Inlet/Outlet Bond Graph links.

### 3.1. 0D Model of the Boiler

**Figure 5:**
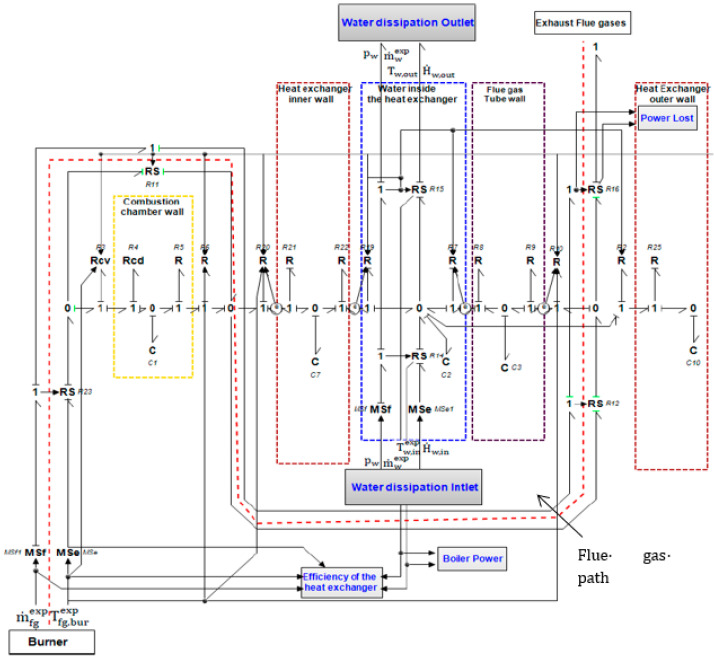
0D model of the boiler.

The 0D dynamic model of the boiler is shown in Figure 5 with all the boiler subsystems (burner, combustion chamber, heat exchanger). The time variation of mass flow rate and temperature of both water and flue gas is considered. As input conditions, the experimental flue gas temperature $T_{\mathrm{fg},\mathrm{bur}}^{\exp}\;$ in the burner and the experimental mass flow rate of the flue gas ${\dot{m}}_{\mathrm{fg}}^{\exp}\;$ are introduced as time files. The water-flue gas heat exchanger is also modelled by providing the experimental water mass flow rate ${\dot{m}}_{w}^{\exp}$ and the experimental inlet water temperature ${\;T}_{w,\mathrm{in}}^{\exp}$ as input conditions. The model is therefore stimulated with real limit conditions and then validated with other experimental measurement obtained at the same time than these limit conditions values. This method improves the validation of the model.

The storage and/or removal of thermal energy in the walls in the different zones of the boiler (refractory concrete, walls of the combustion chamber and walls of the heat exchanger) are modeled by the following law:

(8) $${\overset{•}{Q}}_{\mathrm{wall},i}=m_{\mathrm{wall},i}\times c_{\mathrm{wall},i}\times \frac{{\mathrm{dT}}_{\mathrm{wall},i}}{\mathrm{dt}}=\sum \emptyset_{\mathrm{diss}}$$

With:

${\dot{Q}}_{\mathrm{wall},i}$: heat flux stored in the wall of the system i (W).

$\emptyset_{\mathrm{diss}}\;$: dissipative fluxes between wall and flue gas (W).

m_wall,i_: wall mass of the system i (kg).

c_wall,i_: wall specific heat of the system i (J/kg^−1^.K^−1^).

This expression is traduced to a ‘C’ element (C_1_, C_7_, C_3_, C_10_) (Figure 5) in the bond graph formalism because the flux is a function of the derivative of the effort:
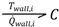


The causality applied to these ‘C’ elements is always a flux causality because at the beginning of the simulation, it is the temperatures (efforts) that are known and then allow calculation of dissipative heat fluxes (conductive, convective and radiative ones). So, in these differential equations solving, the value of temperatures of the next time step are obtained from the integration of the flux balance at the current time.

(9) $$T_{\mathrm{wall},i}\left(t\right)=\frac{1}{m_{\mathrm{wall}}{}_{,i}\times C_{\mathrm{wall},i}}\int_{0}^{t}{\overset{•}{Q}}_{\mathrm{wall},i}\mathrm{dt}$$

where ${\dot{Q}}_{\mathrm{wall},i}$ is calculated from a heat flow balance between all dissipative fluxes $\emptyset_{\mathrm{diss}}$ (Equation (8)) and therefore obtained with a ‘0′ junction centered in the wall.

**Figure 6:**
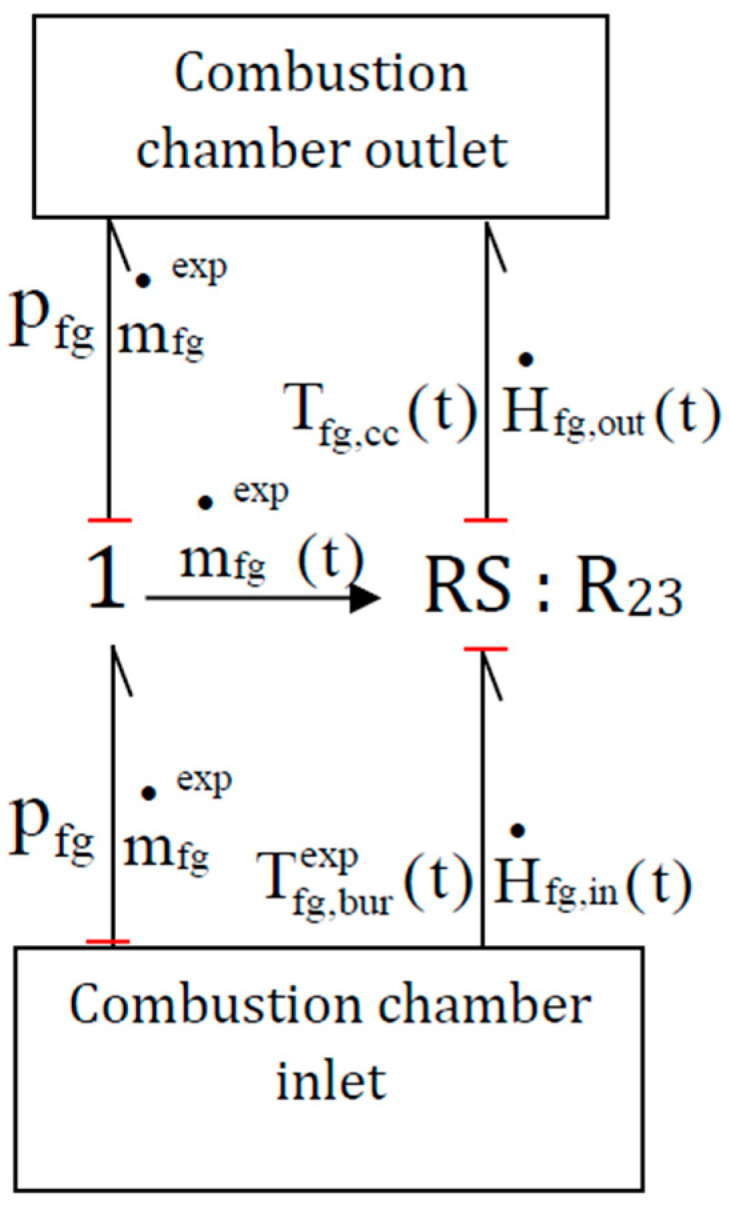
RS element example.

The dynamic behavior of the boiler depends on the interaction between the both hydraulic and thermal systems. The ‘RS’ elements (R_11_, R_12_, R_16_, R_18_ and R_23_) have been used to couple them in order to calculate enthalpy flux (10) from inlet temperature and mass flow rate. The power input of each RS elements is defined with an effort causality which means that the temperature value (effort) of thermal power input is knows at the start of each calculation step. RS elements then calculate the enthalpy flux, which is necessary for each flux balance carried out by the zero junctions on the path of the flue gas labelled by the dashed red line. The enthalpy flux of flue gas at the inlet and outlet of each ‘RS’ element ${\dot{H}}_{\mathrm{fg},\mathrm{in}\;}$ and ${\dot{H}}_{\mathrm{fg},\mathrm{out}\;}$ (Figure 6) is calculated with the following Equations (10) and (11) and the same hypothesis than the Equation (1):

(10) $${\overset{•}{H}}_{\mathrm{fg},\mathrm{in}}\left(t\right)={\overset{•}{m}}_{\mathrm{fg}}^{\exp}\left(t\right).\left[c_{p}\left(T_{\mathrm{fg},\mathrm{in}}\right).\left(T_{\mathrm{fg},\mathrm{in}}\left(t\right)-T_{\mathrm{fg}}^{\mathrm{ref}}\right)+\Delta H_{\mathrm{ref}}^{0}\right]$$

(11) $${\overset{•}{H}}_{\mathrm{fg},\mathrm{out}}(t)={\overset{•}{H}}_{\mathrm{fg},\mathrm{in}}\left(t\right)$$

With: 

${\dot{H}}_{\mathrm{fg},\mathrm{in}}\left(t\right)$: calculated flue gas enthalpy flux at the RS-element inlet (W), equal to ${\dot{H}}_{\mathrm{fg},\mathrm{bur}}$

${\dot{H}}_{\mathrm{fg},\mathrm{out}}\left(t\right)$: calculated flue gas enthalpy flux at the RS-element outlet (W), equal to ${\dot{H}}_{\mathrm{fg},\mathrm{bur}}$

$T_{\mathrm{fg},\mathrm{bur}}^{\exp}\left(t\right)$: experimental temperature in the burner (K)

$T_{\mathrm{fg},\mathrm{cc}}\left(t\right)$: calculated flue gas temperature (K) imported from the following ‘0′ junction in the combustion chamber.

${\dot{m}}_{\mathrm{fg}}^{\exp}\left(t\right)$: experimental flue gas mass flow rate (kg.s^−1^).

$P_{\mathrm{fg}}$: experimental pressure in the boiler (Pa) supposed constant because pressure losses are low and not easy to model in 0D due to the complexity of the geometry.

The Figure 6 illustrates one of the ‘RS’ elements. This one is located between the burner outlet and the combustion chamber.

The thermal transfers by conduction and convection are modeled by equations of the following form:

(12) $${\overset{•}{Q}}_{\mathrm{diss}}\left(t\right)=\frac{1}{R_{\mathrm{th}}}.\;\Delta T\left(t\right)$$

With:

${\dot{Q}}_{\mathrm{diss}}\left(t\right)$: dissipative flux (W).

$R_{th}$: thermal resistor (K.W^−1^).

ΔT(t): temperature difference (K).

Here, the flux ${\dot{Q}}_{\mathrm{diss}}\left(t\right)$ directly depends on the effort ΔT(t) (not derivative link) whether it’s linear or not. In this case the ‘R’ element is used in the bond graph formalism:
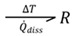


ΔT(t) is obtained with a ‘1’ junction which consist in an effort balance and then can calculate the temperature difference.

R_4_, R_5_, R_21_, R_22_, R_8_, R_9_ and R_25_ quantify the conductive exchanges through the walls. In a cylinder, the thermal conductive resistance R_cd_ is given by:

(13) $$R_{\mathrm{cd},i}=\frac{\ln\left(\frac{r_{2,i}}{r_{1,i}}\right)}{2\pi .\lambda_{i}.H_{i}}$$

With: 

$r_{2,i}$: outside radius of the system i (m).

$r_{1,i}$: inside radius of the system i (m).

λ_i_: thermal conductivity of the system i (W.m^−1^.K^−1^).

H_i_: height for the system i (m).

R_3_, R_6_, R_20_, R_10_, and R_2_ deal with the convective transfers between the flue gas and the different walls of the boiler. They are calculated from the convective resistance R_cv_:

(14) $$R_{\mathrm{cv},i}=\frac{1}{h_{g,i}{.S}_{i}}$$

With:

h_g,i_: global thermal transfer coefficient of the system i (W.m^−2^.K^−1^).

S_i_: exchange surface of the system i (m^2^).

**Figure 7:**
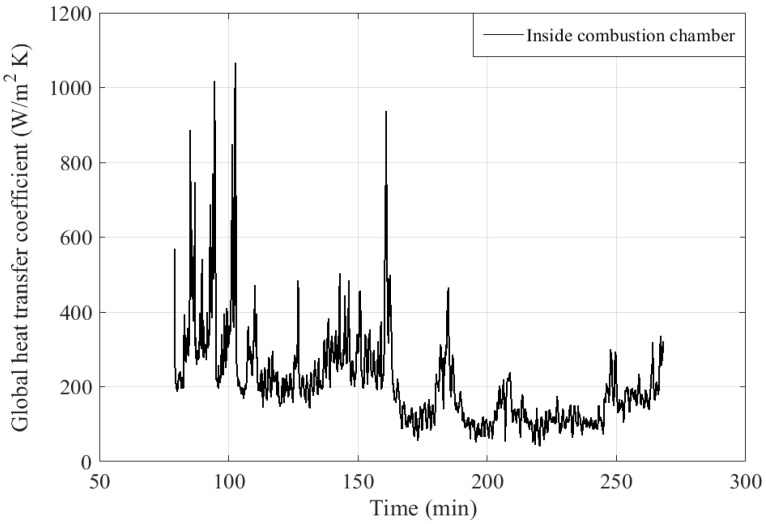
Global thermal transfer coefficient inside the combustion chamber.

The global thermal transfer coefficients h_g,i_, including convective and radiative effects, for the different geometrical configurations in the boiler are obtained according to a first stage of simulation by inverse method. Indeed, the radiative effects of the flame or the gases with the walls are complex to model in 0D. This method is based on the energy balances presented in Section 2.2 for each zone. Heat fluxes are calculated by using experimental wall and flue gas temperatures as well as experimental water temperatures (and calculated temperatures by the dynamic 0D model when the experimental measurement is not available). These experimental temperature values are introduced into the model at each calculation time step. They thus allow at each time step to calculate the value of h_g,i_ as illustrated in the following relations (Equation (15)) in order to use it for the calculation of the parietal fluxes in the model. This method allows to obtain temporal evolutions of h_g,i_ coefficients like the one presented in Figure 7 and was implemented only once as a prerequisite to the main simulation. This allowed to determine the global coefficients even in areas where we were unable to place thermocouples probes by using temperatures calculated as close to reality as possible since in places where temperatures were measured, the model took them into account at each time step. This combination of measured and calculated quantities inside a behavioral model is similar to a HIL (Hardware in the Loop) process.

By example, the variation of the flue gas enthalpy flux between the inlet and the outlet of the combustion chamber $\Delta {\dot{H}}_{\mathrm{fg},\mathrm{cc}}\left(t\right)$ is calculated at each time step. The global heat flux exchanged between the flue gas and the combustion chamber wall ${\dot{Q}}_{\mathrm{fg},\mathrm{cc}}\left(t\right)$ is calculated at each time step also. By performing a balance between the two heat fluxes, the value of global thermal transfer coefficient is deduced for each time step (Equation (15)). The time evolution of a global thermal transfer coefficient, including radiative and convective transfers, near the inside combustion chamber is shown in Figure 7. The peaks observed are due to the low temperature difference between the flue gas and the wall of the combustion chamber. With the induced errors on the global thermal transfer coefficients higher than 2000 W.m^−2^.K^−1^), they cause discrepancies in the calculation carried out by the dynamic model. This adds complexity to the choice of the computation scheme.

(15) $$\begin{array}{c} \Delta {\overset{•}{H}}_{\mathrm{fg},\mathrm{cc}}(t)={\overset{•}{m}}_{\mathrm{fg}}^{\exp}(t)\cdot (\underset{\mathrm{cc}\;\mathrm{outlet}}{\underset{︸}{c_{p,\mathrm{fg}}\left(T_{\mathrm{fg},\mathrm{cc}}^{\exp}\left(t\right)\right){.T}_{\mathrm{fg},\mathrm{cc}}^{\exp}\left(t\right)}}-\underset{\mathrm{cc}\;\mathrm{intlet}}{\underset{︸}{c_{p,\mathrm{fg}}\left(T_{\mathrm{fg},\mathrm{bur}}^{\exp}\left(t\right)\right){.T}_{\mathrm{fg},\mathrm{bur}}^{\exp}\left(t\right)}})\\ {\overset{•}{Q}}_{\mathrm{fg},\mathrm{cc}}\left(t\right)=h_{g,\mathrm{cc}}.S_{\mathrm{cc}}\;\left(T_{\mathrm{fg},\mathrm{cc}}^{\exp}\left(t\right)-T_{\mathrm{wall},\mathrm{cc}}^{\exp}\left(t\right)\right)\\ h_{g,\mathrm{cc}}\left(t\right)=\frac{\Delta {\overset{•}{H}}_{\mathrm{fg},\mathrm{cc}}\left(t\right)}{S_{\mathrm{cc}}\;\left(T_{\mathrm{fg},\mathrm{cc}}^{\exp}\left(t\right)-T_{\mathrm{wall},\mathrm{cc}}^{\exp}\left(t\right)\right)} \end{array}$$

With:

$\Delta {\dot{H}}_{\mathrm{fg},\mathrm{cc}}$: variation of the flue gas enthalpy flux between the inlet and the outlet of the combustion chamber (W)

${\dot{Q}}_{\mathrm{fg},\mathrm{cc}}$: global heat flux exchanged between the flue gas and the combustion chamber wall (W)

c_pfg_: flue gas specific heat at constant pressure (J.kg^−1^.K^−1^).

S_cc_: combustion chamber exchange surface (m^2^).

h_g,cc_: global thermal transfer coefficient for the inner wall of the combustion chamber (W.m^−2^.K^−1^).

$T_{\mathrm{wall},\mathrm{cc}}^{\exp}$: experimental temperature of the inner wall of the combustion chamber (K).

**Figure 8:**
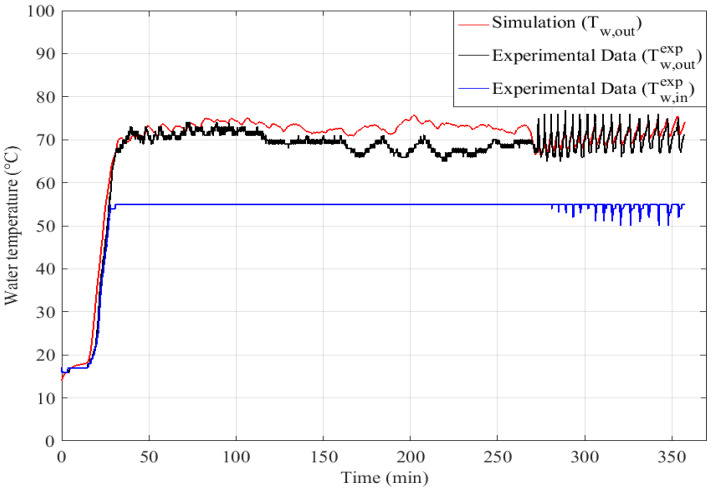
Comparison of experimental and calculated water temperatures at the outlet of the water-flue gas heat exchanger.

**Figure 9:**
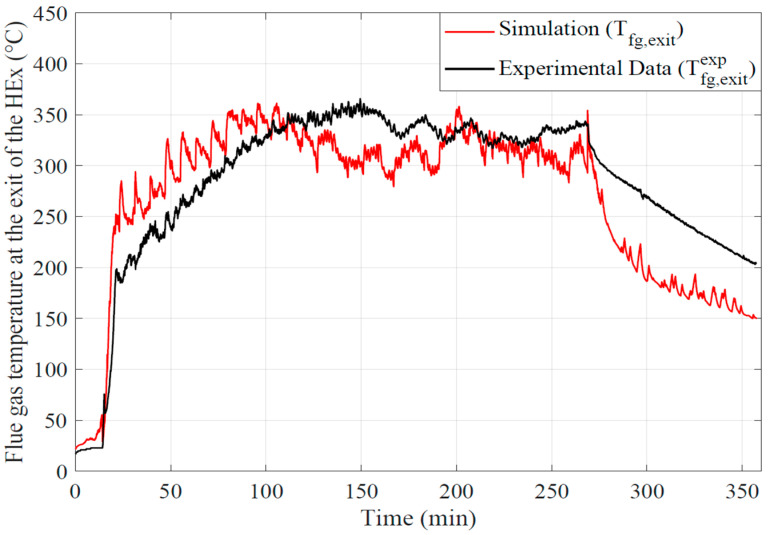
Comparison of experimental and calculated flue gas temperature at the outlet of the flue gas tubes.

For this example, apart from the peaks mentioned above, an average value of h_g_ = 200 W.m^−2^.K^−1^ has been selected. The results presented in Figure 8 and Figure 9 show that this approximation leads to some errors in the calculated water and flue gas temperatures. Indeed, we could identify here two operating regimes:

- h_g_ = 300 W.m^−2^.K^−1^ for *t* = 0–170 min
- h_g_ = 100 W.m^−2^.K^−1^ for *t* = 170–250 min.

**Figure 10:**
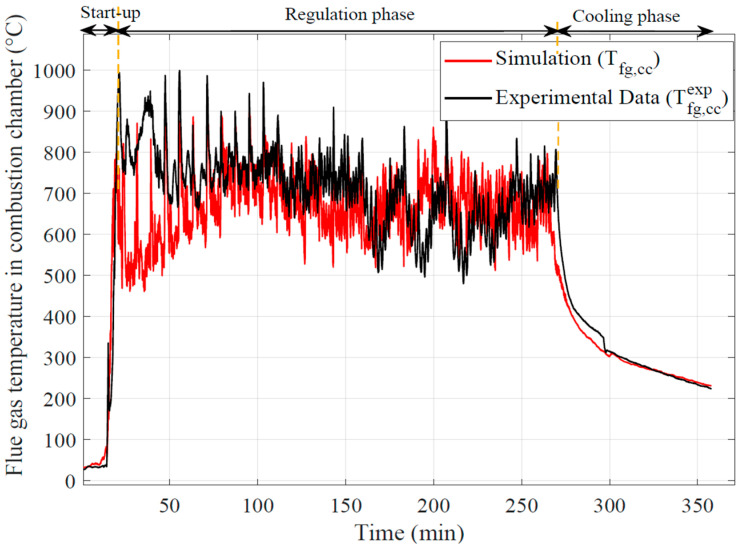
Comparison of experimental and calculated flue gas temperatures in the combustion chamber.

These two operating regimes can be identified in Figure 10, the combustion is continuous until *t* = 170 min and then an operating cycle is set up as presented in Figure 2. The choice of only one value for the global coefficient generates an under estimation of the transfers on the first phase and an over estimation on the second one as it can be noticed in Figure 8 and Figure 9.

Nevertheless, the change of operating regime is difficult to take into account here in an automated way without modeling the pellet supply mechanism and their subsequent combustion.

In order to differentiate radiative and convective heat exchanges in the boiler, the convective heat fluxes are calculated using the Newton’s law and the convective coefficients from Equation (16). Knowing Reynolds number as well as Prandtl, Nusselt numbers was determined from the semi-empirical correlations of Dittus-Boelter [37] and Gnielinski [38], adapted to the studied configurations. Nusselt number then allows to calculate the convective exchange coefficient within the geometric configurations remaining inside the boiler. Table 2 includes the semi-empirical correlations used to calculate Nusselt number.

(16) $$\mathrm{Nu}=\frac{h{.D}_{h}}{\lambda_{\mathrm{fg}}}$$

With:

D_h_: hydraulic diameter (m).

λ_fg_: flue gas thermal conductivity (W.m^−1^.K^−1^).

h: convective coefficient (W.m^−2^.K^−1^).

Finally, the calculation of the water temperature at each instant is obtained by a flux balance represented by the area inside the blue dotted lines in Figure 5.

The flux balance consists of the algebraic sum of the enthalpy input/output fluxes calculated by the ‘RS’ elements (respectively ‘R_14_’ and ‘R_15_’) with the convective heat fluxes calculated with the thermal resistances ‘R_2_’, ‘R_7_’ and ‘R_19_’. The water temperature is then calculated from the integration of the flux balance performed in the ‘C_2_’ element.

### 3.2. Flue Gas Thermodynamic Properties

To take into account the variation of the thermodynamic properties of the flue gas in the boiler, correlations have been used for each property as a function of the temperature.

In this section, all the correlations used to calculate the thermodynamic properties are detailed. The properties are: density ρ_fg_ from the perfect gas law with R = 8.314 J/mol^−1^/K^−1^, thermal conductivity λ_fg_, dynamic viscosity μ_fg_, and specific heat c_pfg_ of the flue gas resulting from the combustion of the pellets. The mass fraction used to calculate some properties is evaluated from the molar fraction deduced from Equation (17). The correlation versus temperature of c_pfg_ is introduced in the 0D model to calculate enthalpy flux. The other thermodynamic properties correlation are used to calculate Reynolds and Prandtl numbers in order to deduce the convective transfer coefficients introduced above.

The thermodynamic properties of flue gas are obtained by adding the properties of each species multiplied by the corresponding molar or specific fractions. The mixture of these species is given by the combustion reaction.

From an elementary analysis, the chemical formulation of pellet is C_36_._725_H_71_._6_O_30_._475_. Then, the combustion reaction of pellets in air is given as below:

(17) $$\begin{array}{l} C_{36.725}H_{71.6}O_{30.475}+39.388\left(1+e_{\mathrm{air}}\right)\left(O_{2}+3.76N_{2}\right)\\ \;\to 36.725{\;\mathrm{CO}}_{2}+35.8{\;H}_{2}O+39.388.e_{\mathrm{air}}.O_{2}+148.097\left(1+e_{\mathrm{air}}\right)N_{2} \end{array}$$

With e_air_ the air excess.

As discussed before, it is assumed that the pressure in the boiler remains constant and equal to the atmospheric pressure and the specific fractions of the combustion products also remain constant during the boiler cycles and in the different zones. Knowing that the boiler operates with an air excess of 80% (e_air_ = 0.8), the correlations used are presented in Table 3.

### 3.3. Solver Scheme

The Bond Graph method uses a system of algebraic-differential equations to describe the dynamic of the modeled system. The accuracy of the dynamic model is based on the choice of the computation scheme used to efficiently solve these differential equations. The resolution scheme used in our model is the Runge-Kutta fourth order formula (RK4) [42,43] which is a particulate case of Runge-Kutta method. This method is recommended when the required accuracy is very high but it requires more CPU time than simpler methods (for this study about 5 min). This method is based on the iteration principle, i.e., an estimation of the solution is calculated from the previous solution. The principle is to approach the next value y_n+1_ at time t_n+1_ by the current value y_n_ obtained at time t_n_ combined with a function taking into account the iteration step (δ) and the estimated slope. The slope is obtained by the weighted average of four slopes (k_1_, k_2_, k_3_ and k_4_), where each slope is the product of the iteration step and an estimated slope. The slope is specified by the function F on the right side of the differential equation [44,45].

The following problem is then considered:

(18) $$\dot{y}=F\left(t,y\right)\;\mathrm{with}\;y_{0}{=f(t}_{0})\to y=f(t)$$

From a known initial condition, the RK4 method is given by the equation:

(19) $$y_{n+1}=y_{n}+\frac{\delta }{6}\left(k_{1}+2k_{2}+2k_{3}+k_{4}\right)+ο\left(\delta^{5}\right)$$

(20) $${\delta =t}_{n+1}{-t}_{n},\;1<n<N$$

where

(21) $$k_{1}=F\left(t_{n}{,y}_{n}\right)$$

(22) $$k_{2}=F\left(t_{n}+\frac{\delta }{2}{,y}_{n}+\frac{k_{1}}{2}\right)$$

(23) $$k_{3}=F\left(t_{n}+\frac{\delta }{2}{,y}_{n}+\frac{k_{2}}{2}\right)$$

(24) $$k_{4}=F\left(t_{n}+\delta ,y_{n}{+k}_{3}\right)$$

The RK4 method is of order 4, this means the error committed at each step is of the order of δ^5^, whereas the total accumulated error is of the order of δ^4^.

## 4. Results and Discussion

The experimental results are discussed to characterize the boiler operation. They are then compared to the simulations. In order to validate the model, measurements of the flue gas temperature profiles in the combustion chamber and at the heat exchanger outlet as well as measurements of water temperature at boiler outlet are carried out.

The dynamic model input data are the experimental flue gas temperature in the burner ($T_{\mathrm{fg},\mathrm{bur}}^{\exp})$, the experimental water temperature at the boiler inlet ($T_{w,\mathrm{in}}^{\exp}$), the experimental flue gas mass flow rate$\;({\dot{m}}_{\mathrm{fg}}^{\exp})$ and the experimental water mass flow rate (${\dot{m}}_{w}^{\exp}$).

The thermal behaviors of the flue gas in the boiler and the water in the heat exchanger are investigated.

From the analysis of the flue gas temperature profiles in the combustion chamber (Figure 10), sudden and fast temperature changes occur during the boiler start-up due to the uncontrolled combustion of a large mass of pellets during this step. Before the combustion start, pellets are heated during several minutes (about 15 min) with an air heater. During the entire control phase (regulation phase between 30–270 min), the flue gas temperatures remain very high and display fluctuations. Then, they decrease progressively during the cooling phase (270–355 min). The fast fluctuations observed are due to the quantities of pellets supplied every 20 s. These fluctuations are also observed on the temperature profiles calculated from the 0D model as a consequence of the limit condition that is an experimental measurement of the flue gas temperature in the burner. We can note that a notable difference exists between the calculated temperature and the one measured during the beginning of the combustion phase (just after the start-up jump). This difference is undoubtedly linked to the fact that the quantity of pellets burning in this phase is very important (accumulation before combustion start), also the gasification is such that the combustion continues in the upper combustion chamber (above the burner zone). The model only integrates the combustion in the burner and therefore does not integrate this excess of heat release in the combustion chamber area.

This problem would have been the same using a combustion model of the pellets in the burner area. It would be necessary to separate the combustion in the 2 zones (burner and combustion chamber) and thus to find a key of distribution of the combustible gases in each zone. This key is not easy to find because the problem is related to unsteady 3D aero thermochemical phenomena.

The water temperature at the heat exchanger outlet is also examined. As noted for the flue gas temperature, a drastic increase of the water temperature can be observed during the start-up phase. During this phase (0–30 min), the water circulates in closed circuit until to reach a temperature of 325 K (Figure 8). This process is imposed by the mixing valve (3-way valve) resulting in a significant increase of the water temperature. After this step, the hot water is redirected to the cooling circuit. The fluctuations observed during the cooling phase are due to the intermittent operation of the water pump to maintain as long as possible the boiler body closed to the operating conditions if the boiler needs to be restarted.

Here, the differences between the calculated and measured values are not significant. Differences of 5 °C are nevertheless noted in the cyclic operation zone (170–250 min), this is perhaps linked to the overestimation of the global exchange coefficients in this operation mode as mentioned at the end of Section 3.1.

The instantaneous evolutions of the experimental and calculated flue gas temperature at the outlet of the flue gas tube of the water heat exchanger are plotted in Figure 10. The flue gas temperature at the flue gas tube outlet has the same evolution as in the combustion chamber. Nevertheless fluctuations are filtered by the thermal inertia of the different parts of the boiler along the flue gas path. The temperature of the flue gas remains relatively high at outlet of the tubes (~573 K).

As mentioned at the end of Section 3.1, here the under estimation of the wall global exchange coefficients on the stabilized phase and the under estimation on the cyclic phase is notable. In the stabilized phase, the underestimation of the thermal wall fluxes limits the thermal dissipation of the gases and thus also the reduction of their temperature. In the cyclic phase, the overestimation of the fluxes increases abnormally the wall transfers and reduces the flue gas temperature. Significant temperature differences remain during the cooling phase of the boiler. 

**Figure 11:**
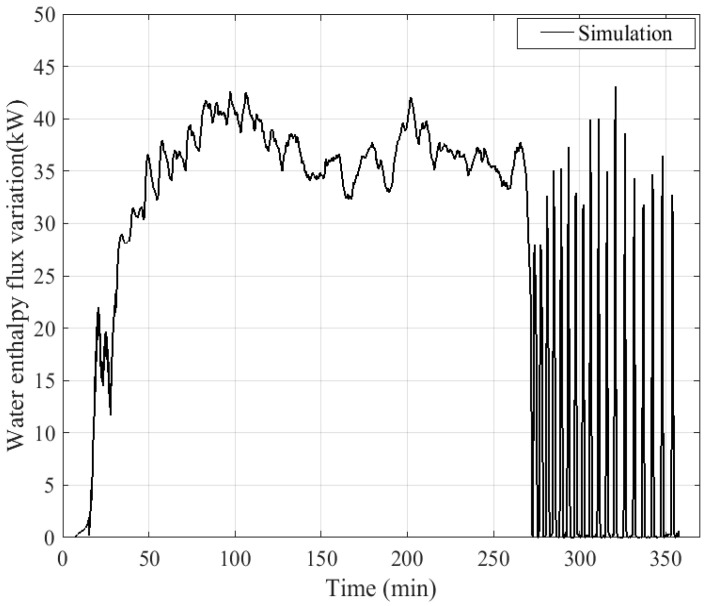
Variation of water enthalpy flux.

The time evolution of the water enthalpy flux variation between inlet and outlet, calculated by the dynamic model, is plotted in Figure 11. The heat flux drops to zero when the pump stops.

The instantaneous thermal power delivered to the water can be calculated as:

(25) $$\Delta {\overset{•}{H}}_{w}\left(t\right)={\overset{•}{m}}_{w}^{\exp}\left(c_{w}\left(T_{w,\mathrm{out}}\right){.T}_{w,\mathrm{out}}{-c}_{w}\left(T_{w,\mathrm{in}}^{\exp}\right){.T}_{w,\mathrm{in}}^{\exp}\right)$$

With:

$\Delta {\dot{H}}_{w}\left(t\right)$: water enthalpy flux variation (W).

$T_{w,\mathrm{in}}^{\exp\;}\;$: experimental water temperature at the heat exchanger inlet (K).

$T_{w,\mathrm{out}}$: water temperature at the heat exchanger outlet calculated by the dynamic model (K).

c_w_: water specific heat at an average temperature of 328 K (4183/kg^−1^.K^−1^).

The amount of heat flux transmitted to the water remains very low during the start-up phase of the boiler and then increases drastically after the start of the combustion. After the start of combustion, an increase of the heat transmitted to the water can be observed between 30 and 50 min in Figure 11. This progression exists because some heat from combustion is first accumulated by the metal walls inside the boiler before being completely transferred to the water when the walls reach an established thermal regime. Considering only the regulation phase represented by the period from the combustion start time (30 min) to the boiler shutdown (270 min), the heat flux transmitted from the flue gas to the water of the heat exchanger is quite stable and close to 37 kW. The fluctuations observed on the flue gas temperature during the cyclic phase are well absorbed by the inertia of the walls and the water. After the boiler shutdown and during the cooling phase, intermittent operation of the pump is observed. When the pump is shut down, the walls of the heat exchanger transmit heat to the volume of water became motionless in the heat exchanger, which explains the peaks of enthalpy flux as soon as the pump is started up again.

From the dynamic model of the boiler, it is also possible to calculate its efficiency, lost power, heat flux stored by the walls and released by combustion in the burner.

According to the manufacturer, the boiler must have an average efficiency of 85% under nominal operating conditions (thermal power of 30 kW). In this study, the duty cycle (PWM) of the pellet supply screw was modified to increase the power of the boiler in order to saturate the downstream thermal load and thus create thermal control cycles suitable to unsteady operating conditions.

The efficiency can be defined as the ratio of the enthalpy flux variation of the water heat exchanger and the heat flux released from pellet combustion:

(26) $$\eta \left(t\right)=\frac{{\Delta \dot{H}}_{w}\left(t\right)}{{\dot{H}}_{\mathrm{fg},\mathrm{bur}}\left(t\right)}$$

**Figure 12:**
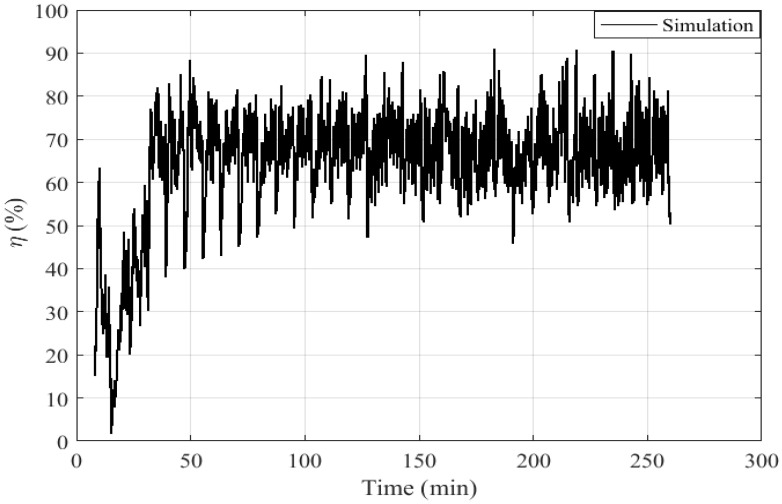
Calculated boiler efficiency.

The instantaneous evolution of the efficiency calculated by the model at each moment t is presented in Figure 12. Its evolution is drastically affected by the fluctuations of flue gas temperature. The boiler has an average efficiency of 67.5%. This low efficiency value is not surprising because the overpower generated in our test case cannot be fully absorbed by the capacity of the gas-water exchanger of the boiler. The thermal power of the boiler is nevertheless increased (37 kW instead of 30 kW).

**Figure 13:**
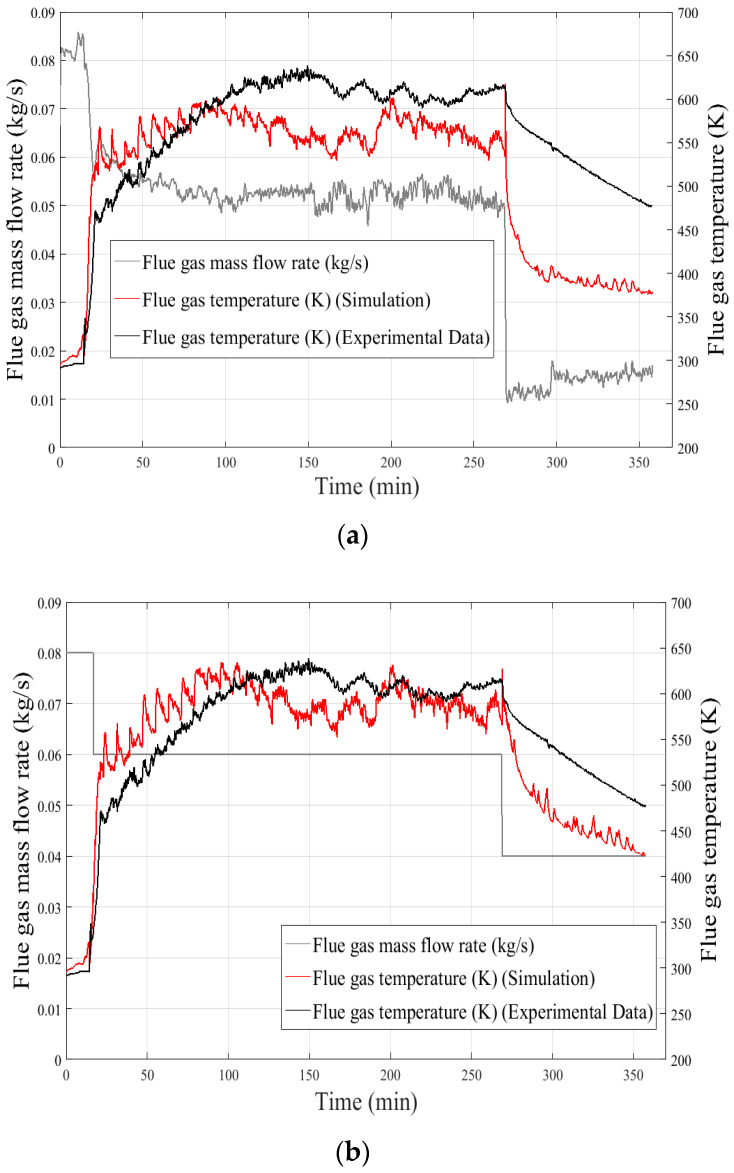
Influence of the flue gas mass flow rate at the flue gas outlet on the 0D model response. (**a**): With experimental flue gas mass flow rate, (**b**): With a flue gas mass flow corrected for each boiler operating phase.

Several parameters impacting the response of the 0D model can be highlighted. For example, an influence on the thermal behavior of the flue gas with the mass flow rate can be distinguished at the tube outlet. As the experimental flue gas mass flow rate is used as input condition, during the cooling phase a significant discrepancy between the evolution of the calculated and experimental flue gas temperatures is recorded as shown in Figure 13a.

This discrepancy can be explained by a low value of flue gas flow rates during the cooling phase (Figure 13a) according to the accuracy of the measurement chain (Pitot tube associated with a micromanometer and a thermocouple), which involves a maximum error of 40%. By adjusting the mass flow rate value of the flue gas, staying within the uncertainty range of the flowmeter, a clear improvement of the model response is observed (Figure 13b).

A heat flux balance in four zones of the boiler is performed by using the equations introduced in Section 2.2 and allows to compare the radiative and convective heat flux. The convective heat flux is calculated by using the convective coefficients obtained from the semi-empirical correlations given in Table 2 and the total heat flux ϕ_tot_ is obtained by using the global thermal transfer coefficients calculated from the inverse method by carrying out flux balances inside the boiler using Equations (3)–(6), previously introduced in Section 2.2. The radiative flux can be then deduced from the total heat flux, knowing the convective heat flux.

Due to the presence of large temperature gradients in the boiler, combustion products such as water vapor (H_2_O), carbon dioxide (CO_2_) and soot particles radiate significantly. Radiation is the dominant thermal transfer in the boiler and must be compared to the total thermal transfers (Table 4).

## 5. Conclusions

A 0D dynamic modeling of a domestic biomass boiler of low power was developed by using Bond Graph formalism that allows to represent the coupled multi-physical phenomena, to study the thermal transfers between the different fluids during the transient operating phases, to evaluate the energy performances of the boiler and to take into account the variability of the heat production. The local evolution of the state variables is much less detailed than with CFD modeling but the dependencies of one zone with another are better taken into account with a 0D dynamic modeling. A biomass combustion model was not developed in this study but the combustion reaction of pellets in air allowed to calculate the thermodynamic properties of the flue gas in the boiler used in the 0D model. This model based on mass and energy balances was validated with experimental results, in particular the flue gas temperature in several locations of the boiler and the water temperature at the heat exchanger outlet. Some experimental data and 0D modeling at each time step of the calculation were coupled. The thermal transfers between the flue gas and the water circulating inside the heat exchanger and between these two fluids and the boiler structures were simulated. The experimental results showed a dependence of the evolution of the flue gas temperature in the combustion chamber as a function of the quantity of pellets supplied, according to the thermal cycle of the boiler. This directly affects the operating conditions of the boiler and generates important temperature fluctuations in the combustion chamber, which could significantly affect the operation of a hot air machine in the case of a conversion into a micro cogeneration unit. Indeed in this case, the air-gas exchanger of such an installation would be located in the zone where the temperature is the highest and thus closest to the flame. A calculation of the global thermal transfer coefficients by inverse method was done in the subsystems of the boiler. A good agreement between the experimental measurements and the simulation has been found and the origins of the differences have been identified, such as the excess of heat release in the combustion chamber above the burner zone not integrated in the model. It has been shown that the boiler has an average efficiency of 67.5% and the radiation is the dominant thermal transfer in the boiler by reaching 97.6% of the total thermal transfers inside the combustion chamber. The 0D dynamic model of the boiler during the operating phases allows not only to evaluate its energy performances but also to highlight the boiler components where the thermal transfers must be optimized.

The modeling of pellet combustion using a heat release law adapted to solid biomass combustion associated with an efficient identification of the pellet mass flow rate will make it possible to improve this model and make it independent of experimental boundary conditions. The radiative transfers being preponderant but difficult to model in 0D for mutual exchanges between gases, particles and walls, a detection of the different combustion phases and this according to the presence or not of flame in each of the zones would allow to better parameterize the global exchange coefficients which moreover will be able to be identified with the help of the proposed inverse method. The modeling methodology developed will allow the study of a complex unit, such as a CHP plant by coupling the different models for each component.

## Figures and Tables

**Table 1:** Review of energy system modeling.

Reference	Device	Study	Power	Main Objective
Strzalka et al. [8]	Biomass grate furnace	Mathematical modeling	6 kW	Model-based optimization of control strategies of grate furnaces.
Li et al. [9]	Biomass boiler	Thermodynamic modeling		Conventional exergy analysis and advanced exergy analysis of a real biomass boiler.
Kang et al. [10]	Biomass boiler	Experimental investigation	24 kW	Evaluation of the performances of a domestic wood pellet boiler.
Gómez et al. [11]	Biomass domestic boiler	CFD modeling	27 kW	Simulation of the boiler operation under transient conditions. The effect of the parameters influencing the combustion process has been studied.
Ziviani et al. [17]	ORC system	Dynamic modeling (AMESim)		Progress and challenges related to the operation of ORC (Organic Rankine Cycle) systems.
Féniès et al. [18]	Stirling engine	Theoretical modeling and experimental study	18 W	Establishment of two models, thermal and electrical, and study of the influence of dead volume, the natural frequency of mechanical oscillations and thermal conduction between the hot and cold sides for engine optimization.
Abdulmoneim et al. [22]	Thermal power generation station	Dynamic modeling (Bond Graph)		Modeling of hybrid power plant: pump, boiler, economizer, evaporator, super heater, drum and pipe.
Creyx et al. [19]	Ericsson engine	Dynamic modeling (Bond Graph)		Dynamic model of the expansion cylinder of an open Joule cycle Ericsson engine.
Ould-Bouamama et al. [30]	Chemical reactor	Dynamic modeling (Bond Graph)		Modeling of a chemical reactor for monitoring.
Sandberg et al. [33]	Biomass boiler	Dynamic modeling	157 MW	Biomass boiler dynamic model.
Persson et al. [34]	Biomass boiler and stove	Dynamic modeling (TRNSYS)	10 kW	Development and validation of a dynamic boiler/pellet stove model based on experimental measurements.

**Table 2:** Semi-empirical correlations used for the calculation of Nusselt number.

Location	Flow Configuration	Correlations	Valid Range
Combustion chamber and flue gas tubes [37]	Inside a cylinder	Nu=0.023ReDh0.8Pr0.4(1+(DhH)0.7)	0.7 ≤ Pr ≤ 120 104 ≤ ReDh ≤ 1.2 10^5^ $2\;\leq \;D_{h}$ ≤ 20
Passage between the combustion chamber and inner wall of the heat exchanger [38]	Inside an annular duct–fixed walls	Nu=0.023ReDh0.8Pr0.4(r2/r1)0.14	0.7 < Pr < 100 ReDh > 2000

**Table 3:** Correlations for the calculation of the flue gas thermodynamic properties (i = CO_2_, H_2_O, O_2_, N_2_)**.** The constants A, B, C, D and E were fixed for each species and for each property.

Flue Gas Thermodynamic Properties	Correlations	Units	Temperature Range (K)	Min–Max
Density	$\rho_{i}=\frac{PM_{i}}{RT}$ $\rho_{fg}(T_{fg})={\left(\sum_{i}\frac{y_{i}}{\rho_{i}(T_{fg})}\right)}^{-1};{\;y}_{i}=\frac{m_{i}}{m_{tot}};$	kg.m^−3^	298–1500	0.23–1.22
Thermal conductivity [39]	$\lambda_{i}=A+BT_{fg}+CT_{fg}^{2}+DT_{fg}^{3}$ $\lambda_{fg}=\frac{\sum_{i}x_{i}\lambda_{i}M_{i}^{1/3}}{\sum_{i}x_{i}M_{i}^{1/3}},{\;x}_{i}=\frac{n_{i}}{n_{tot}}$	W.m^−1^.K^−1^	298–1500	2.32 10^−2^–8.65 10^−2^
Dynamic viscosity [40]	$\mu_{i}(T)=A+BT+CT^{2}+DT^{3}$ $\mu_{fg}(T_{fg})=\frac{\sum_{i}x_{i}\mu_{i}(T_{fg})\sqrt{M_{i}}}{\sum_{i}x_{i}\sqrt{M_{i}}}$	Pa.s^−1^	298–1500	1.711 10^−5^–5.42 10^−5^
Specific heat [41]	$c_{p,i}(T_{fg})=A+BT_{fg}+CT_{fg}^{2}+DT_{fg}^{3}+E/T_{fg}^{2}$ $c_{p,}{}_{fg}(T)=\sum_{i}y_{i}c_{p,}{}_{i}(T_{fg})$	J.kg^−1^.K^−1^	298–1500	1090–1374

**Table 4:** Heat flux balance.

Location	ϕ_rad_/ϕ_tot_ (%)
Inside the combustion chamber	97.6
Outside the combustion chamber (annular passage)	96.8
Inside the heat exchanger (flue gas side)	96.1
Inside the flue gas pipes	95.6

## Data Availability

Not applicable.

## Nomenclature

c_p,fg_(T)	Flue gas specific heat (J.kg^−1^.K^−1^) at constant pressure, function of temperature T
c_w_	Water specific heat (J.kg^−1^.K^−1^)
c_wall_	Wall specific heat (J.kg^−1^.K^−1^)
D_h_	Hydraulic diameter (m)
H	Combustion chamber height (m)
h_g_	Global thermal transfer coefficient (W.m^−2^.K^−1^)
${\dot{H}}_{\mathrm{fg},[\mathrm{area}]}$	Enthalpy flux of the flue gas in a specific area (W)
${\dot{H}}_{w,[\mathrm{area}]}$	Enthalpy flux of the water in a specific area (W)
k_1_	First slope of Runge-Kutta fourth order formula
k_2_	Second slope of Runge-Kutta fourth order formula
k_3_	Third slope of Runge-Kutta fourth order formula
k_4_	Fourth slope of Runge-Kutta fourth order formula
m_wall_	Wall mass (kg)
${\dot{m}}_{\mathrm{fg}}^{\exp}$	Experimental flue gas mass flow rate (kg.s^−^^1^)
${\dot{m}}_{\mathrm{pellets}}^{\exp}$	Experimental pellets mass flow rate (kg.s^−^^1^))
${\dot{m}}_{w}^{\exp}$	Experimental water mass flow rate (kg.s^−^^1^)
p_w_	Water pressure (Pa)
p_fg_	Flue gas pressure (Pa)
${\dot{Q}}_{w}$	Heat flux transferred to the water (W)
${\dot{Q}}_{\mathrm{wall}}$	Heat flux stored in the boiler structure (W)
${\dot{Q}}_{\mathrm{fg},\mathrm{cc}}$	Convective heat flux exchanged between the flue gas and the combustion chamber wall (W)
${\dot{\;Q}}_{\mathrm{wall},\mathrm{HEx}}$	Heat flux stored in the heat exchanger wall (W)
${\dot{\;Q}}_{\mathrm{wall},\mathrm{tub}}$	Heat flux stored in the walls of the flue gas tubes (W)
$r_{1}$	Inside radius (m)
$r_{2}$	Outside radius (m)
R_cd_	Conduction resistance (K.W^−1^)
R_cv_	Convective resistance (K.W^−1^)
S	Exchange surface (m^2^)
t	Time (s)
T_amb_	Ambient temperature (K)
${\;T}_{\mathrm{fg},\mathrm{bot}}^{\exp}$	Experimental flue gas temperature at the bottom of the heat exchanger (K)
$T_{\mathrm{fg},\mathrm{bur}}^{\exp}$	Experimental flue gas temperature in the burner (K)
$T_{\mathrm{fg},\mathrm{cc}}^{\exp}$	Experimental flue gas temperature in the combustion chamber (K)
$T_{\mathrm{fg},\mathrm{exh}}^{\exp}$	Experimental flue gas temperature in the chimney (boiler exhaust) (K)
$T_{\mathrm{fg},\mathrm{exit}}^{\exp}$	Experimental flue gas temperature at the flue gas tubes outlet (K)
$T_{w,\mathrm{in}}^{\exp}$	Experimental water temperature at the inlet of the heat exchanger (K)
$T_{w,\mathrm{out}}^{\exp}$	Experimental water temperature at the outlet of the heat exchanger (K)
$T_{\mathrm{fg},\mathrm{in}}$	Calculated flue gas temperature at the RS-element inlet (K)
$T_{\mathrm{fg},\mathrm{out}}$	Calculated flue gas temperature at the RS-element outlet (K)
$T_{\mathrm{fg},\mathrm{top}}^{\exp}$	Experimental flue gas temperature at the top of the combustion chamber (K)
$T_{\mathrm{wall},\mathrm{inner}}^{\exp}$	Experimental temperature of the inner wall of the combustion chamber (K)
$T_{\mathrm{wall},\mathrm{outer}}^{\exp}$	Experimental temperature of the outer wall of the combustion chamber (K)
T_wall_	Calculated wall temperature (K)
**Subscripts**	
air	Air
amb	Ambient
bur	Burner
bot	Bottom
cc	Combustion chamber
cd	Conductive
cv	Convective
exit	Exit
exh	Exhaust
fg	Flue gas
g	Global
HEx	Heat Exchanger
in	Inlet
rad	Radiative
tub	Tube
top	Top
tot	Total
out	Outlet
w	Water
wall	Wall
**Superscript**	
exp	Experimental Value
**Greek symbols**	
Δ	Variation of thermodynamic quantity
λ_i_	Wall thermal conductivity (W.m^−^^1^.K^−^^1^)
λ_fg_	Flue gas thermal conductivity (W.m^−1^.K^−1^)
ρ_fg_	Flue gas density (kg.m^−3^)
μ_fg_	Flue gas dynamic viscosity (Pa.s)
η	Boiler efficiency (%)
δ	Iteration step of Runge-Kutta fourth order formula
**Dimensionless numbers**	
Re	Reynolds number
Pr	Prandtl number
Nu	Nusselt number
